# mHealth Series: Text messaging data collection of infant and young child feeding practice in rural China – a feasibility study

**DOI:** 10.7189/jogh.03.020403

**Published:** 2013-12

**Authors:** Xiaozhen Du, Wei Wang, Michelle Helena van Velthoven, Li Chen, Robert W. Scherpbier, Yanfeng Zhang, Qiong Wu, Ye Li, Xiuqin Rao, Josip Car

**Affiliations:** * Joint first authorship; 1 Department of Integrated Early Childhood Development, Capital Institute of Pediatrics, Beijing, China; 2 Global eHealth Unit, Department of Primary Care and Public Health, Imperial College London, London, United Kingdom; 3 Section of Health and Nutrition, Water, Environment and Sanitation, UNICEF China, Beijing, China

## Abstract

**Background:**

Face–to–face interviews by trained field workers are commonly used in household surveys. However, this data collection method is labor–intensive, time–consuming, expensive, prone to interviewer and recall bias and not easily scalable to increase sample representativeness.

**Objective:**

To explore the feasibility of using text messaging to collect information on infant and young child feeding practice in rural China.

**Methods:**

Our study was part of a clustered randomized controlled trial that recruited 591 mothers of children aged 12 to 29 months in rural China. We used the test–retest method: first we collected data through face–to–face interviews and then through text messages. We asked the same five questions on standard infant and young child feeding indicators for both methods and asked caregivers how they fed their children yesterday. We assessed the response rate of the text messaging method and compared data agreement of the two methods.

**Finding:**

In the text messaging survey, the response rate for the first question and the completion rate were 56.5% and 48.7%, respectively. Data agreement between the two methods was excellent for whether the baby was breastfed yesterday (question 1) (kappa, κ = 0.81), moderate for the times of drinking infant formula, fresh milk or yoghurt yesterday (question 2) (intraclass correlation coefficient, ICC = 0.46) and whether iron fortified food or iron supplement was consumed (question 3) (κ = 0.44), and poor for 24–hour dietary recall (question 4) (ICC = 0.13) and times of eating solid and semi–solid food yesterday (question 5) (ICC = 0.06). There was no significant difference in data agreement between the two surveys at different time intervals. For infant and young child feeding indicators from both surveys, continued breastfeeding at 1 year (*P* = 1.000), continued breastfeeding at 2 years (*P* = 0.688) and minimum meal frequency (*P* = 0.056) were not significantly different, whereas minimum dietary diversity, minimum accepted diet and consumption of iron–rich or iron fortified foods were significantly different (*P* < 0.001).

**Conclusions:**

The response rate for our text messaging survey was moderate compared to response rate of other studies using text messaging method and the data agreement between the two methods varied for different survey questions and infant and young child feeding indicators. Future research is needed to increase the response rate and improve data validity of text messaging data collection.

Malnutrition of infants and young children is highly prevalent in low– and middle–income countries and closely linked, either directly or indirectly, to major causes of child deaths [1]. In 2012, 12.6% of Chinese children younger than five years were underweight and 9.4% were stunted [2]. Inadequate breastfeeding and complementary feeding are the major causes of undernutrition in young children [3]. Infant and young child feeding (IYCF) practice in China is suboptimal: the exclusive breastfeeding rate for infants younger than 6 months was only 27.6%; the proportion of infants aged 6–9 months who received complementary feeding was 43.3%; and the proportion of children aged 12–15 months who received continued breastfeeding was only 37.0% [4]. Therefore, there is an urgent need to improve IYCF practices in China. Accurate and timely measurements of IYCF indicators are essential to inform decision makers, program managers and donors to make evidence–based decisions.

Evidence–based maternal, newborn and child health (MNCH) interventions can improve the processes and outcomes of health care when appropriately implemented, and therefore contribute to reduction of the death for children under five [5]. The MNCH interventions should achieve high levels of coverage in children who need them to maximize the effectiveness [6]. High–quality measurements of intervention coverage are crucial to track progress and make evidence–based decisions [7]. MNCH coverage data in most low– and middle–income countries are mainly generated through household surveys, such as Demographic and Health Surveys (DHS) [7,8] and Multiple Indicator Cluster Surveys (MICS) [9]. In China, the nationally representative data on infant and young child feeding are mainly collected from the National Health Services Survey and National Nutritional and Health Survey. These two surveys are generally carried out every five years and face–to–face interviews with caregivers are the standard method for data collection [4,10].

However, face–to–face data collection is labor–intensive, time consuming and expensive [11]. Therefore, new methods need to be explored to overcome shortcomings of the face–to–face method and text messaging could be an innovative way of data collection due to the rapid increase in mobile phone use [12]. In 2013, there were almost as many mobile phone subscriptions as people in the world, with more than half in the Asia–Pacific region (3.5 billion out of 6.8 billion total subscriptions) [13]. In China, there were more than 1.1 billion mobile phone subscriptions as of May 2013 [14] and text messaging is very commonly used. Text messaging could be used to measure MNCH coverage in China [15]. Text messaging has a significant potential to reduce the cost, and interviewer and recall bias and to increase the sample size and sample representativeness of household surveys [15]. However, there are also many challenges for text messaging data collection and a series of studies need to be conducted before this method could be used [15]. Data validity and response rate are the two major issues that need to be addressed. The MNCH coverage indicators that could be collected by text messaging method include antenatal care, delivery and postnatal care, infant and young child feeding, immunization and common childhood diseases [16]. This study will explore the feasibility of using text messaging to collect data on IYCF practices.

## METHODS

We used test–retest method to compare two data collection methods for 24–hour recall of infant and young child feeding: face–to–face vs text messaging. The current feasibility study was part of a larger study, a clustered randomized controlled trial, aiming to evaluate the effectiveness of QQ (a popular Chinese instant messaging program) as a channel to deliver IYCF information on, in reducing anemia prevalence in Zhao County, Hebei Province, China. For the trial, we collected data on IYCF practices using face–to–face method in the end line survey of the trial. To conduct the current feasibility study, we collected the same data again from the same participants by text messaging method (QQ was only used in the trial for delivering IYCF information to mothers of children, not for data collection in this feasibility study). On one day, we first conducted face–to–face interviews with mothers during the day, and then asked them to reply to our text messages that had the same questions in the evening after 18:00.

### Study setting

We carried out this study in seven townships, Zhao County, Hebei Province, China (detailed description of the study setting can be found elsewhere [17,18].

### Participants

Prior to this study, we conducted a baseline survey for the clustered randomized controlled trial (cRCT) in January 2013 and recruited caregivers of all children aged 6–23 months in the seven townships. Caregivers were eligible for this comparison study if they: 1) took part in the baseline survey of the trial; 2) were mothers of the child (our previous experiences indicated that grandparents were generally unable to reply text messages and fathers usually did not know the child's feeding behavior); 3) had mobile phones and were able to reply to text messages; 4) were willing to participate. We excluded mothers who completed face to face survey after 18:00, because our text messaging survey started at 18:00.

### Training of interviewers

Interviewers for the face to face survey were medical students from Hebei Medical University and were trained for two days on the survey procedures. The training consisted of communication skills, explanation of questionnaires, demonstration, role plays, field practice, and group discussion throughout the course. Interviewers were encouraged to ask questions when they encountered any problems. Inter– and intra–interviewer reliability for completing survey instruments after the training was assessed using a standardized role play by two supervisors. The reliability was over 95% in all measurements.

### Recruitment

A doctor from Zhao County Maternal and Child Health Hospital was our local guide, and helped us to connect with local township doctors and village doctors. We obtained the name list of both children and their mothers with mobile phone numbers of mothers from the baseline study. Using the name list, the county doctor contacted township doctors before the study started to arrange an appropriate time for the interviews. The township doctors then informed village doctors of the accurate time for interviewing and asked them to recruit mothers on the name list to come to the village clinics. When mothers came to clinics, the supervisors first checked their phone numbers. If mothers had changed their numbers, supervisors recorded the new numbers and then informed a team member (XD) who sent text messages in Beijing to update numbers. The interviewers obtained written informed consent for both face to face and text messaging surveys. After the face–to–face survey, the interviewers reminded mothers to reply to the text messages before 12:00 am at night, and explain in which format they had to reply to the text messages. We gave each mother a towel of 5 Yuan (¥) (equal to US$ 0.81) for her time in the face–to–face study and we paid ¥ 5 mobile phone credit to mothers who completed text messaging survey.

### Questionnaires and pilot study

This study included two survey questionnaires: (i) the traditional face to face survey and (ii) the text messaging survey.

For the face–to–face survey, we used the WHO questionnaires for assessing IYCF practices, which had been adapted to local context in Zhao County and been used in our previous studies [16,17].

For the text messaging survey, our study team first discussed how to adapt the seven questions that were used in face–to–face survey, so that they had similar content, but more understandable in text messaging format and easier to reply to. All seven questions were then tested in a pilot study. We selected a convenience sample of 217 caregivers in Shahedian Township (not included in the feasibility study), Zhao County, and after obtaining informed consent, we sent seven text messaging questions to them. For the pilot study, 105 (48.4%) out of 217 participants responded to our first question and 26 (12.0%) out of 217 completed all seven questions. After the pilot, we conducted interviews with mothers in Shahedian Township to collect their feedback and advices on our text messaging questions.

For each question, we asked mothers what the questions meant and whether there were any problems in understanding our text messaging questions. We also encouraged them to offer their advice to make the questions easier to understand.

We planned to interview mothers who replied in the pilot study and those who never received our text messaging questions, because mothers who replied may have been more familiar with our text messaging questions and may have encountered problems when responding. This way we could obtain more insight in how to revise our text messaging questions based on their previous experiences. For those who did not receive message questions, we sent each text messaging question via an iPhone 4 and asked them to reply to us during the interview. We checked their reply messages immediately and if we found that there were any unclear answerers, we asked the mothers why they replied like this and whether there was anything else that was unclear in the text message.

We interviewed 18 mothers in Shahedian Township: nine of them had replied to all text messages and nine had not received our text messages (not included in the feasibility study). We revised our text messaging questions according to mothers’ feedback, and the main changes were: 1) reduced the total number of text messages (from 7 to 5); 2) changed the order of text messages; 3) adapted the content of text messages (see Online Supplementary Document(Online Supplementary Document)). The final text messaging survey consisted of nine messages: three introduction messages (Text Message 1–3) which did not need a reply, five survey question messages (Text Message 4–8) from which six IYCF indicators can be calculated, and one “Thank you” message. Detailed description of the text messages is shown in **Box 1**.

Box 1 **Text messaging survey contents**

**Table:** 

**Text message1** Hello! This is Zhao County Maternal and Child Health Hospital and Capital Institute of Pediatrics. We have tested hemoglobin in your child earlier today. Now we would like to ask you some questions about feeding of your child through text messages. **Text message 2** We will send 5 text message questions simultaneously to you at 18:00, please reply to each text message separately. See next message for reply formats. If you answer all 5 questions, you will receive 5 Yuan mobile phone credit within 2 weeks. **Text message 3** Please respond with the following format: question number + your answer. **Text message 4 (Q1)** Was your child breastfed yesterday during the day or at night(from 6:00 am yesterday to 6:00 am today)?Please respond: the number of this question + your answer to this question. **Text message 5 (Q2)** How many times did your child drink infant formula, fresh milk, or yoghurt yesterday during the day or at night (from 6:00 am yesterday to 6:00 am today) totally? Please respond: the number of this question + your answer to this question. **Text message 6 (Q3)** Yesterday, during the day or night (from 6:00 am yesterday to 6:00 am today), did your child consume iron fortified infant formula, iron fortified rice, iron fortified noodles, or any iron supplement (including liquids, powders or sprinkles)? Please respond: the number of this question + which one did your child consume. **Text message 7 (Q4)** Please recall the order of time and list everything (including meals and snacks) that your child ate or drank from 6:00 am yesterday to 6:00 am today, whether at home or outside the home. Please respond: the number of this question + your answer to this question. **Text message 8 (Q5)** From 6:00 am yesterday to 6:00 am today, how many times did your child eat solid, semisolid, or soft foods other than liquids? All thick foods should be included, eg, noodles, steamed bread, cookies, bread, meat, fruits, vegetables, eggs and thick porridge, etc. Only one or two bites of foods, and liquids (water, thin soup and drinks) should not be included. Please respond: the number of this question + your answer to this question. **Text message 9** This is the end of the survey. Thank you very much for your participation! You will receive ¥ 5 mobile credit within two weeks.

### Data collection and entry process

**Face–to–face survey.** Village doctors asked eligible mothers to gather in village clinics for the interviews. Interviewers recorded mothers’ responses with a smart phone. The smart phones automatically recorded the time of completed questionnaires and uploaded the data into an excel database. The advantages of using smart phone for data collection can be found in our former study [17].

**Text messaging survey.** We sent text messages to mothers who took part in the face–to–face survey. A team member first sent three introduction text messages to mothers in order to introduce ourselves, tell mothers how to correctly reply to text messages and inform them of the ¥ 5 (equal to US$ 0.81) mobile phone credit for completing this survey. Then we sent five text messaging questions simultaneously at 18:00. We numbered all five questions and asked mothers to add in their reply messages the same number of each question. A text message was sent as a reminder at 19:00 and 20:00 if mothers had not replied to all text messages. Finally we sent a “thank you” message to those who completed text messaging survey and told them they would receive ¥ 5 (equal to US$ 0.81) mobile credit for their fees of replying text messages and time consumption.

We used a Chinese text messaging system (Shangjibao,商机宝) for sending and receiving text messages. We asked customer–service workers of the system to contact us if there was any problem with sending and receiving messages.

Two of the team member (XD,XR) transferred answers of text messaging responding to numbers independently in order to create text messaging database, disagreements were solved by consulting a third team member who was experienced with nutritional surveys (QW).

**Outcomes.** The primary outcomes of our study were the response rate of text messaging survey and data agreement between the two methods. The secondary outcome was the difference in IYCF indicators between the responders and non–responders of text messages.

**Response rate.** In the text messaging survey, we defined and reported the response rate in two ways: (i) response rate to the first question; proportion of mothers who responded to the first question, and (ii) completion rate; proportion of mothers who responded to all five questions.

**Data agreement.** We compared the answers to each question from the face–to–face survey and text messaging survey by the same individual mother. For test–retest method, ideally there should be an appropriate time interval between the two tests, but there were no early literature for reference in our study. We compared data agreement of the two surveys at different time intervals. We first divided mothers who replied to our text messages into two groups by a specific time point: before 11:45 group and after 11:45 group because at this time point the number of mothers in each group was similar. We then calculated time interval between the two methods using text messaging sending time (18:00) subtracting the completion time of the face–to–face survey. In addition, we also compared the IYCF indicators calculated from face–to–face survey and text messaging survey between the two groups. More detailed information on the calculation of selected IYCF indicators can be found in Online Supplementary Document(Online Supplementary Document).

Difference in IYCF indicators between the responders and non–responders to the text messages

We calculated and compared IYCF indicators of responders and non–responders of text messaging based on the face–to–face survey.

**Data analysis.** We used chi–square test and Mann-Whitney U/Wilcoxon W (MWU/WW) test to compare the characteristics of responders and non–responders of text messaging survey. In addition, we assessed data agreement by kappa (κ) values (simple κ for categorical variable), intraclass correlation coefficient (ICC, for quantitative variables) and percentages of the same answers in both methods. We used McNemar’s test for binary outcomes and extended McNemar’s test [19] for nominal variables to detect differences between survey methods in IYCF indicators. We used SAS 9.1 (SAS Institute, Cary, NC, USA) for the analysis and we considered a P–value less than 0.05 as statistically significant.

## Results

Among 788 caregivers who participated in the end line survey of the clustered randomized controlled trial, 591 mothers were eligible for our text messaging survey. A total of 197 caregivers were excluded because they were not mothers (n = 97), completed the face–to–face survey after 18:00 (n = 13), had no mobile phones (n = 52), had twins (n = 6), or we failed to send text messages (n = 29) (field supervisors forgot to inform the text message sender). **Table 1** lists the demographic characteristics of mothers and their children. The demographic characteristics between the responders and non–responders were similar.

**Table 1:** Demographic characteristics of children and their mothers

Variable	Total (No., %)	Responders of text messages* (No., %)	Nonresponders of text messages* (No., %)	Statistics	P value
**Children**					
**Gender:**					
Boy	302 (51.1)	145 (50.4)	157 (51.8)	χ^2^ = 0.13	0.721
Girl	289 (48.9)	143 (49.6)	146 (48.2)		
**Age in months:**					
12–23	451 (76.3)	215 (74.7)	236 (77.9)	χ^2^ = 0.86	0.355
24–29	140 (23.7)	73 (25.3)	67 (22.1)		
**Mothers**					
**Median age in years** (Q1–Q3)	25 (24–28)	25 (24–28)	25 (23–29)	MWU/WW Z = –0. 83	0.410
**Years of education:**					
0–9	488(82.6)	237 (82.3)	251 (82.8)	Fisher exact test	0.933
10–18	100(16.9)	50 (17.4)	50 (16.5)		
Unknown	3(0.5)	1 (0.3)	2 (0.7)		
**Occupation:**					
Home	541 (91.5)	263 (91.3)	278 (91.8)	χ^2^ = 0.04	0.851
Work	50 (8.5)	25 (8.7)	25 (8.2)		
**Mother is the primary caregiver:**					
Yes	492 (83.3)	246 (85.4)	246 (81.2)	χ^2^ = 1.89	0.169
No	99 (16.8)	42 (14.6)	57 (18.8)		

MWU/WW – Mann-Whitney U/Wilcoxon test

*We defined responders as mothers who replied to all five text messages, and non–responders as mothers who did not reply to all five text messages.

### Response rate

**Figure 1:**
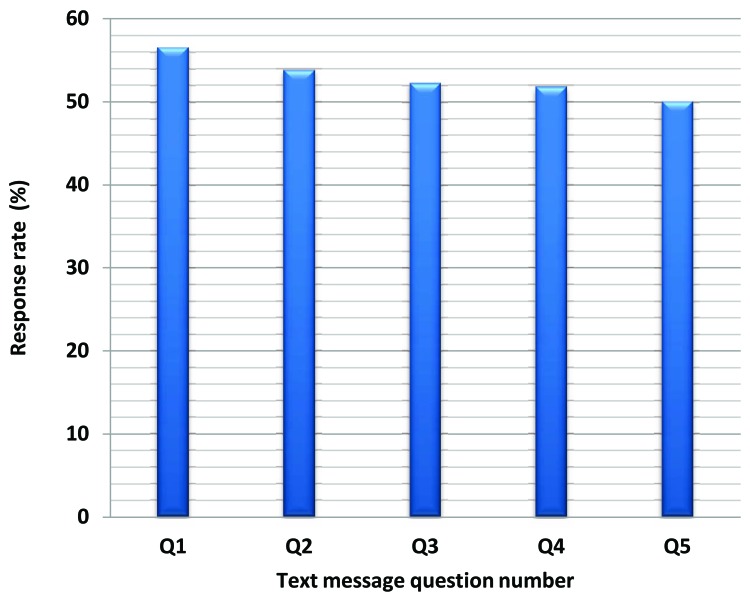
Response rate of each question for text messaging survey.

**Figure 1** shows the response rate of each question for the text messaging survey. The response rate of the first question was 56.5% and the completion rate was 48.7% respectively. There was a slightly decreased trend (*P* = 0.022) in response rates.

**Figure 2:**
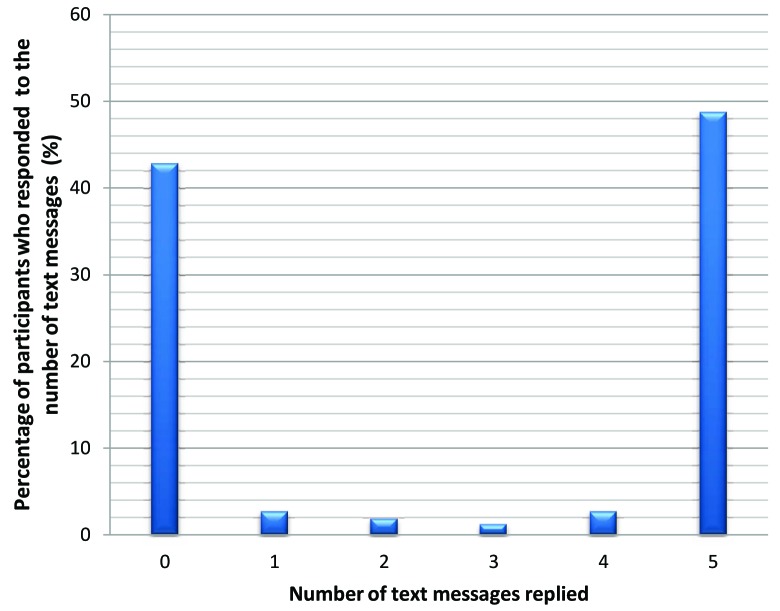
Percentage of mothers who replied to different numbers of text messages.

**Figure 2** indicates the percentage of mothers who replied different numbers of text messages. There were 253 (42.8%) out of 591 mothers who never responded and 288 (48.7%) out of 591 mothers who completed our text messaging survey. Very few mothers 50 (8.5%) out of 591 who responded did not complete the text message survey.

### Data agreement

**Table 2** shows that agreement between the two methods for all five questions varied to a great extent. Agreement was excellent for the first question (κ = 0.81, 95% confidence interval 0.75–0.86), moderate for the second (ICC = 0.46, 95% confidence interval 0.37–0.55) and third questions (κ = 0.44 95% confidence interval 0.30–0.58), and poor for the fourth (κ = 0.13, 95% confidence interval 0.08–0.18 for minimum food frequency; κ ranged from 0.02 to 0.36 for all the seven food categories) and fifth questions (ICC = 0.06, 95% confidence interval –0.05–0.17).

**Table 2:** Data agreement between the two methods

No. of text messaging questions	No. of pairs	Face–to–face (No., %)	SMS (No., %)	Total agreement (No., %)	McNemar test	P value	κ/ICC(95% CI)
Q1: Breastfed yesterday*	338	175 (51.8)	169 (50.0)	303 (89.6)	3.50‡	0.174	0.81 (0.75–0.86)
Q2: Times of drinking milk† yesterday	301	0 (0–2)	1 (0–2)	0 (–1–0)	–3090	<0.001	0.46 (0.37–0.55)
Q3: Iron–rich food and supplement yesterday*	224	171 (76.3)	175 (78.1)	180 (80.4)	0.36	0.546	0.44 (0.30–0.58)
Q4: Dietary recall:							
Grains, roots and tubers*	338	335 (99.1)	285 (84.3)	284 (84.0)	46.3	<0.001	0.02 (–0.04–0.08)
Legumes and nuts*	338	102 (30.2)	19 (5.6)	245 (72.5)	74.08	<0.001	0.15 (0.06–0.24)
Dairy products*	338	152 (45.0)	217 (64.2)	227 (67.2)	38.06	<0.001	0.36 (0.27–0.45)
Flesh foods*	338	196 (58.0)	68 (20.1)	196 (58.0)	115.38	<0.001	0.23 (0.16–0.30)
Eggs*	338	293 (86.7)	196 (58.0)	227 (67.2)	84.77	<0.001	0.26 (0.17–0.34)
Vitamin–A rich fruits and vegetables*	338	270 (79.9)	73 (21.6)	135 (39.9)	191.18	<0.001	0.10 (0.06–0.15)
Other fruits and vegetables*	338	283 (83.7)	152 (45.0)	199 (58.9)	116.74	<0.001	0.19 (0.12–0.26)
No. of food categories reported†	338	5 (4–6)	3 (2–4)	2 (1–3)	21420.5	<0.001	0.41 (0.32–0.49)
Minimum of diversity*	338	290 (85.8)	121 (33.1)	161 (47.6)	161.36	<0.001	0.13 (0.08–0.18)
Q5: Times of having solid and semi–solid food yesterday†	330	4 (3–4)	6 (4–7)	–2 (–3–0)	–16145	<0.001	0.06 (–0.05–0.17)

95% CI – 95% confidence interval

*Simple kappa (κ).

†Report median (interquartile range, IQR) for face–to–face, SMS and the difference (face–to–face way–SMS way), use pairwise Wilcoxon test, and intraclass correlation coefficient (ICC).

‡Extended McNemar test.

The earliest time at which a mother completed the face–to–face survey was 7:55 in the morning and the latest time was 17:53 in the afternoon. We used the median time point of 11:45 in the morning to divide mothers into 2 groups. The text messaging survey started at 18:00. We calculated that the median time interval between the face–to–face and text messaging survey was 8.1 hours (Q1–Q3, 7.30–8.78) for the before 11:45 group and 3.4 hours (Q1–Q3, 2.1–5.3) for the after 11:45 group. **Table 3** shows the data agreement for the two groups. There were overlaps for the 95% confidence interval of the κ and ICC in data agreement for all the indicators between the two groups.

**Table 3:** Data agreement at different time intervals of the two surveys*

No. of text message questions	Total			Before 11:45				After 11:45		
	**No. of pairs**	**Total agreement (%)**	**κ/ICC (95% CI)**	**No. of pairs**	**Total agreement (%)**		**κ/ICC**	**No. of pairs**	**Total agreement (%)**	**κ/ICC(95% CI)**
Q1: Breastfed yesterday†	338	303 (89.6)	0.81 (0.75–0.86)	167	151 (90.4)		0.82 (0.74–0.89)	167	149 (89.2)	0.80 (0.73–0.88)
Q2: Times of drinking milk yesterday‡	301	0 (–1–0)	0.46 (0.37–0.55)	149	0 (–1,0)		0.519 (0.39–0.63	149	0 (–1,0)	0.36 (0.22–0.49)
Q3: Iron–rich food and supplement yesterday†	224	180 (80.4)	0.44 (0.30–0.58)	108	120 (81.5)		0.430 (0.22–0.64)	115	92 (80.0)	0.463 (0.28–0.65)
Q4: Dietary recall										
Grains, roots and tubers†	338	284 (84.0)	0.02 (–0.04–0.08)	167		136 (81.4)	–0.11 (–0.034–0.011)	167	145 (89.2)	0.063 (–0.08–0.20)
Legumes and nuts†	338	245 (72.5)	0.15 (0.06–0.24)	167		128 (76.7)	0.16 (0.019–0.30)	167	114 (68.3)	0.14 (0.03–0.25)
Dairy products†	338	227 (67.2)	0.36 (0.27–0.45)	167		114 (68.3)	0.39 (0.26–0.52)	167	110 (65.9)	0.32 (0.18–0.46)
Flesh foods†	338	196 (58.0)	0.23 (0.16–0.30)	167		98 (58.7)	0.23 (0.13–0.34)	167	96 (57.5)	0.23 (0.14–0.33)
Eggs†	338	227 (67.2)	0.26 (0.17–0.34)	167		107 (64.1)	0.24 (0.12–0.36)	167	118 (70.7)	0.273 (0.15–0.40)
Vitamin–A rich fruits and vegetables†	338	135 (39.9)	0.10 (0.06–0.15)	167		68 (40.7)	0.11 (0.06–0.17)	167	67 (40.1)	0.10 (0.04–0.16)
Other fruits and vegetables†	338	199 (58.9)	0.19 (0.12–0.26)	167		97 (58.1)	0.22 (0.11–0.32)	167	92 (55.1)	0.16 (0.07–0.25)
No. of food categories reported‡	338	5 (4–6)	0.41 (0.32–0.49)	167		2(1–3)	0.49 (0.36–0.59)	167	2 (1–3)	0.32 (0.18–0.45)
Min of diversity†	338	161 (47.6)	0.13 (0.08–0.18)	167		81(48.5)	0.16 (0.08–0.23)	167	79(47.31)	0.10 (0.03–0.17)
Q5: Times of having solid and semi–solid food yesterday‡	330	–2 (–3–0)	0.06 (–0.05–0.17)	165		–2(–4–0)	–0.01 (–0.17–0.14)	161	–2(–3–0)	0.17 (0.02–0.32)

95% CI – 95% confidence interval

*There were 6 caregivers interviewers recorded their responses by pen–and–paper, therefore the completed time wasn’t recorded, and we deleted those in this part.

†Simple kappa (κ).

‡Report median (interquartile range, IQR) for face–to–face, SMS and the difference (face–to–face way–SMS way), use pairwise Wilcoxon test, and intraclass correlation coefficient (ICC).

**Table 4** presents IYCF indicators for responders of text messages calculated from face–to–face survey and text messaging survey. There were no significant differences for continued breastfeeding at 1 year (*P* = 1.000), continued breastfeeding at 2 years (*P* = 0.688) and minimum meal frequency (*P* = 0.056). However, the differences for minimum dietary diversity, minimum accepted diet and consumption of iron–rich or iron fortified foods were significant (*P* < 0.001).

**Table 4:** IYCF indicators for responders of text messages based on face–to–face and text messaging surveys

Number of indicators	No. of pairs	Face–to–face survey (%)	Text messaging survey (%)	Comparison	
				**McNemar test**	**P value**
1: Continued breastfeeding at 1 year	72	90.3 (n = 65)	88.9 (n = 64)	0.33	1.000
2: Continued breastfeeding at 2 year	98	41.8 (n = 41)	39.8 (n = 39)	0.67	0.688
3: Minimum meal frequency	217	73.7 (n = 160)	65.9 (n = 143)	3.66	0.056
4: Minimum dietary diversity	222	86.9 (n = 193)	37.8 (n = 84)	103.31	<0.001
5: Minimum accepted diet	215	54.4 (n = 117)	20.9 (n = 45)	52.90	<0.001
6: Consumption of iron–rich or iron fortified foods	225	60.0 (135)	33.3 (n = 75)	40.00	<0.001

### Difference of IYCF indicators between the responders and non–responders of text messages

**Table 5** illustrates IYCF indicators calculated for responders of text messages, non–responders and for all participants based on face–to–face survey database. There was no significant difference between responders and non–responders for all six indicators (P value ranging from 0.139 to 1.000).

**Table 5:** IYCF indicators based on face–to–face survey

Number of indicators	Total		Non–responder of text messages		Responder of text messages		Comparison	
	**No.**	**%**	**No.**	**%**	**No.**	**%**	**χ^2^**	**P value**
1: Continued breastfeeding at 1 year	127	89.8 (n = 114)	55	89.1 (n = 49)	72	90.3 (n = 65)	–*	1.000
2: Continued breastfeeding at 2 year	174	43.7 (n = 76)	76	46.1 (n = 35)	98	41.9 (n = 41)	0.31	0.578
3: Minimum meal frequency	451	74.9 (n = 338)	234	76.1 (n = 178)	217	73.7 (n = 160)	0.32	0.567
4: Minimum dietary diversity	451	84.9 (n = 383)	229	83.0 (n = 190)	222	87.0 (n = 193)	1.39	0.239
5: Minimum accepted diet	451	54.6 (n = 246)	236	55.7 (n = 129)	215	54.4 (n = 117)	0.003	0.959
6: Consumption of iron–rich or iron fortified foods	451	56.5 (n = 255)	226	53.1 (n = 120)	225	60.0 (n = 135)	2.19	0.139

*Fisher exact test.

## DISCUSSION

### Principal result

Our study examined the feasibility of using text messages to collect infant and young child feeding data. The response rate for the first question and the completion rate were 56.5% and 48.7%, respectively. Agreement was excellent for whether the child was breastfed yesterday (Q1), moderate for the times of drinking infant formula, fresh milk or yoghurt (Q2) and whether iron fortified food or iron supplement was consumed (Q3), and poor for 24–hour dietary recall (Q4) and times of eating solid and semi–solid food yesterday (Q5). Data agreement in the 8.1–hour time interval group and 3.4–hour time interval group was the same. Three IYCF indicators calculated from both the two surveys were not significantly different, whereas the other three were significantly different.

**Response rate.** Response rate is crucial for a successful text messaging data collection. Response rates reported in literatures were highly variable, ranging from 15% [20] to 100% [12]. A study evaluating the use of text messaging for infant feeding questions reported a 92.7% response rate in a cohort of women who recently delivered, asking about their current infant feeding practices and future feeding plans through text messaging [21]. The response rate in our study was moderate comparing with other studies on text messaging data collection, but much higher than our former study conducted in the same county, which had a completion rate of 27.9% in text messaging survey (our unpublished data). There were differences in methodology between the current and former study. Some possible reasons may explain the improvement of response rate. First, the supervisor in each team asked or checked the phone number with mothers, while the former study asked the village doctors to do this. Second, we asked interviewers to remind mothers to reply to our text messages, to explain in which format they had to reply to the text messages, and to tell mothers that they received ¥ 5 mobile credit if they replied to all our text messages. Third, we sent all five text messages simultaneously to mothers, while in the former study, we sent text messages separately. Finally, the number of the core text messaging questions was five, whereas seven for the former study.

We found that the proportion of mothers who responded but did not complete the survey was very low (8.5%) compared to the completion rate (48.7%) and the non–response rate (42.8%). Mothers were likely to not reply to any text message, or to reply to all five text message questions. This may indicate that initiation is very important to increase the response rate of a text messaging survey. We also provided 5 Yuan mobile credit to those who completed our survey and this may have been the reason for a high completion rate.

Low response rate of text messaging survey is very common and may reduce the sample representativeness. We compared the demographic characteristics of responders and non–responders of the text messaging survey and found no significant difference. In addition, we calculated the IYCF indicators for responders and non–responders of text messaging survey based on the face–to–face survey database and found that there were no significant differences for all six indicators. This may imply that a low response rate does not necessarily affect the survey results; however, more efforts are definitely needed to dramatically increase the response rate.

**Data agreement and IYCF indicators.** Data validity is an important issue in instrument development and provides information about the quality of measurements [22]. Whitford et al. [21] showed that a text messaging survey had an excellent agreement compared to a telephone interview to collect information on infant feeding. A study comparing telephone interviews and text message data collection for disease symptom reporting also acquired a high degree of agreement [11]. In our study, agreement for the five questions varied hugely. Agreement was excellent for whether the baby was breastfed yesterday, which suggests that this question could be used in future text messaging surveys. The other four questions had moderate to poor agreement, which implies more studies need to be carried out for data validity of these questions.

Some terms in the questionnaires were not easily understood by mothers, such as iron fortified food or iron supplement and solid food or semi–solid food. In face–to–face survey, the well–trained interviewers could explain this to the mothers. However, this was very difficult for the text messaging survey due to limited length and number of text messages. This implies that interviewer–administered face–to–face survey may still be better for questions which are hard to understand, whereas self–administered text messaging survey may have the potential to ask simple questions which require simple answers, such as Q1 in our study (Was your child breastfed yesterday during the day or at night?). We found that the number of food groups reported was significantly higher in face–to–face survey than in the text messaging survey. In the standard procedure for 24h dietary recall in face–to–face survey, the interviewer first asked the caregivers to recall activities and food intake for the child backwards; the interviewers chose the food group on the list based on caregivers' answer. When they finished the recall part, the interviewer went through every food group that the caregiver did not mention and asked whether the child ate that kind of food in the time period one by one. However, in the text message, we only asked the caregivers to self–report the food that the child ate once and this may explain the fewer categories of caregivers reported in the text messaging method

The reported times of eating solid and semi–solid food yesterday was significantly higher via text messaging. Caregivers may have had different views for solid and semi–solid food and overestimated the times that food was eaten. On the other hand, there might be interviewer bias in the face–to–face survey to underestimate the times, because children in this study were 12– to 29–month old and the interviews were likely to assume that the child eat 3–4 times solid and semi–solid food in a day.

In six IYCF indicators, the difference between the two survey methods for continued breastfeeding at 1 year and continued breastfeeding at 2 years were very small and not significant. These two indicators were based on the Q1, which had excellent agreement between the two methods. The difference for minimum meal frequency was large but not significant. The differences for the other three indicators were large and significant, much higher in face–to–face survey than in the text messaging survey. The calculation for these three indicators based on their responses to the fourth question which involved the 24–hour diet recall. Caregivers reported fewer food categories from which their children ate via the text messaging method, which may help to explain the data inconsistency.

In the test–retest study, the choice of time–interval between the two tests was quite arbitrary. We did not define time–interval ahead, but divided them into two groups by the median time in which we completed the face–to–face survey. There was no difference for data agreement for all survey questions between the two groups (group with time interval between the face–to–face and text message survey of 8.1 hours (median) and 3.4 hours (median). Although the agreement for different recall time intervals for low back pain [11], sedentary time [23], and health related quality of life [24] were reported, no study explored a narrower time range for infant and young child feeding practices.

### Strengths and limitations

To our knowledge, this is the first study exploring collection of standard WHO infant and young child feeding indicators by text messaging in rural China. Our study had some limitations. First, though using text messages as a data collection method was time efficient and user friendly, there was still work on coding the content of text messages and on setting up a text messaging database. Further research has to take this into consideration if text messaging method is to be used on a large scale. Second, the way of sending text messages may not be appropriate if there are some questions that needed to be skipped. Third, we did not evaluate mothers’ acceptability of the methods which may have provided insights in the reasons for the fewer food categories they reported via text messaging method. Fourth, we could not validate whether a responder of text messaging survey was the same person who took part in our face–to–face survey. Fifth, old people in rural areas are usually unable to send text messages; therefore, text messaging survey could not be applied to grandparents of children.

## CONCLUSION

Our feasibility study shows that text messaging survey had a moderate response rate compared to other studies on text messaging data collection and data agreement with face–to–face survey varied very much from question to question. Agreement for IYCF indicators calculated from the two methods also varied. Future research is needed to increase the response rate and improve the data validity of text messaging survey before it could be used.
