# Supplementary material for: mHealth Series: Text messaging data collection of infant and young child feeding practice in rural China – a feasibility study
Source: J Glob Health. 2013 Dec;3(2):020403. doi: 10.7189/jogh.03.020403 (PMC3868816; doi:10.7189/jogh.03.020403)
Supplement: Online Supplementary Document [file jogh-03-020403-s001.pdf]

Online Supplementary Document

Du et al. mHealth Series: Text messaging data collection of infant and young child feeding practice in rural China – a feasibility study

1. More detailed information on development of text messages

Table w1. Development of text messages

N of text messages	Face to face survey	Pilot study	Text messaging survey	Reasons and explanations for the changes
1	NA	Hello, caregivers. We would like to ask you 5 questions about breastfeeding and complementary feeding of your child yesterday. And if you answer all the questions, you will receive 5 Yuan recharged on your mobile phone credit within 2 weeks.	Hello! This is Zhao County Maternal and Child Health Hospital and Capital Institute of Pediatrics. We have tested hemoglobin for your child early today. Now we would like to ask you some questions about feeding of your child through text messages.	We decided to mention 'physical examinations' to make mothers recognise us.
2	NA	NA	We will send 5 text message questions simultaneously to you at 18:00, please reply each text message separately. See next message for reply formats. If you answer all 5 questions, you will receive 5 Yuan mobile phone credit within 2 weeks.	We decided to offer detailed information about text messaging sending and mobile phone credit recharged in order to motivate mothers to reply.

3	NA	NA	Please respond with the following format: question number + your answer. e.g. Was your child breastfed yesterday during the day or at night? If yes, you should respond like this: 1st, yes.	We decided to offer detailed information about the responding format to make mothers more understandable.
4	Was your child breastfed yesterday during the day or at night?<br>Yes<br>No<br>Don't know	Was your child breastfed yesterday during the day or at night?	[1st question]Was your child breastfed yesterday during the day or at night (from 6:00 am yesterday to 6:00 am today)?Please respond: the number of this question + your answer to this question.	1) During the cognitive interviews, mothers said that they felt confused when reading 'the day or at night', therefore we decided to change it to exact time interval.<br><br>2) We decided to remove the options because the screens of mothers' mobile phones were not big enough to read complete messages.
5	Now I would like to ask you about some liquids that your child may have had yesterday during the day or at night. Yesterday, during the day or night, did your child consume infant formula, milk or yoghurt? If yes, how many times did your child consume these?	How many times did your child drink following liquids (infant formula, milk, or yoghurt) yesterday during the day or at night totally?	[2nd question]How many times did your child drink infant formula, or fresh milk, or yoghurt yesterday during the day or at night (from 6:00 am yesterday to 6:00 am today) totally? Please respond: the number of this question + your answer to this question.	We decided to change the order (from the 8th to 5th) of this question because mothers suggested that it would be clearer putting the milk-related information behind breastfeeding information.

6	1) Yesterday, during the day or night, did your child consume any [list iron fortified solid, semi-solid or soft foods designed specifically for infants and young children available in the local setting]?<br>2) Yesterday during the day or night, did your child consume any food to which you added a powder or sprinkles like this?[show common type of sprinkles available in survey area.<br>3) Yesterday, during the day or night, did your child consume any [list iron fortified infant/toddler formulas available in the local setting]?<br>Yes	Yesterday, during the day or night, did your child consume iron fortified rice cereal/noodle/formula or anything iron fortified including liquids, powder and sprinkles?	[3rd question]Yesterday, during the day or night(from 6:00 am yesterday to 6:00 am today), did your child consume iron fortified infant formula, or iron fortified rice, or iron fortified noodle, or any iron supplement (including liquids, powders or sprinkles)?Please respond: the number of this question + which one did your child consume.	We decided to add 'iron fortified' as a reminder because some mothers forgot that the noodles should be iron-fortified.

	No			
	Don't know			
7	Please describe everything that your child ate yesterday during the day or night, whether at home or outside the home.	Please describe everything that your child ate from 6am yesterday to 12am yesterday, whether at home or outside the home.	[4th question]Please recall in the order of time and list everything (including meals and snacks) that your child ate or drank from 6:00 am yesterday to 6:00 am today, whether at home or outside the home. Please respond: the number of this question + your answer to this question.	1) We decided to add 'According to chronological order' to make it more similar to the face-to-face survey.
	A. Bread, rice, noodles, or other foods made from grains, including thick grain-based porridge	Please describe everything that your child ate from 12am yesterday to 5pm yesterday, whether at home or outside the home.		2) We decided to merge these 3 questions because there was a decreased trend in response rate when conducted pilot study and mothers thought it was troublesome to respond to 3 similar questions and they were able to reply that in one text message.
	B. Pumpkin, carrots, squash, or sweet potatoes that are yellow or orange inside	Please describe everything that your child ate from 5pm yesterday to 6am today, whether at home or outside the home.		
	C. White potatoes, white yams, manioc, cassava, or any other foods made from roots?			

D. Any dark green leafy vegetables

E. Ripe mangoes, ripe papayas or (Insert other local vitamin A-rich fruits)

F. Any other fruits or vegetables

G. Liver, kidney, heart or other organ meats

H. Any meat, such as beef, pork, lamb, goat, chicken, or duck

I. Eggs

J. Fresh or dried fish, shellfish, or seafood?

K. Any foods made from beans, peas, lentils, or nuts?

L. Cheese, yoghurt, or other milk products

8	Since this time yesterday, how many times did your child eat solid, semisolid, or soft foods other than liquids?	Since this time yesterday, how many times did your child eat solid, semisolid, or soft foods other than liquids?(some examples in Chinese characteristics)	[5th question] From 6:00 am yesterday to 6:00 am today how many times did your child eat solid, semisolid, or soft foods other than liquids? All thick foods should be included, e.g. noodles, steamed bread, cookies, bread, meats, fruits, vegetables, eggs and thick porridge, etc. Only one or two bites of snacks, and liquids (water, thin soup and drinks) should not be included. Please respond: the number of this question + your answer to this question.	NA
9	NA	We have sent 5 text messaging questions to you this evening, asking you about breastfeeding and complementary feeding of your child. Please respond the rest of text message questions you failed to respond and you will receive 5 Yuan mobile credit if you reply all.	NA	NA

10	NA	We have sent 5 text messaging questions to you this evening, asking you about breastfeeding and complementary feeding of your child. Please respond the rest of text message questions you failed to respond and you will receive 5 Yuan mobile credit if you reply all.	NA	NA
11	NA	This is the end of the survey. Thank you very much for participating! You will receive 5 Yuan for participating within two weeks.	NA	NA

NA – not applicable

2. More detailed information on calculation of selected IYCF indicators.

Table w2. Calculation of selected IYCF indicators

IYCF indicators	Calculation of selected IYCF indicators
1: Continue breastfeeding to 1 years	Proportion of children 12-15 month of age were fed (TM1=1) by breast milk.
2: Continue breastfeeding to 2 years	Proportion of children 20-23 month of age were fed (TM1=1) by breast milk.
3: Minimum diet frequency	Proportion of breastfed (TM=1) children 6-23 months of age who receive solid, semi-solid, or soft foods the minimum number of times or more (TM5>=2 in age 183-274 days or TM5>=3 age in 274-730 days) or proportion of non-breastfed children 6-23 months (TM=2) of age who receive solid, semi-solid, or soft foods or milk feeds the minimum number of times or more ((TM2+TM5)>=4) during the previous day.
4: Minimum food diversity	The food group including: 1) Grains, roots and tubers; 2) Legumes and nuts; 3) Dairy products (milk, yoghurt, cheese); 4) Flesh foods (meat, fish, poultry and liver/organ meats); 5) Eggs ;6) Vitamin-A rich fruits and vegetables; 7) Other fruits and vegetables. The coding was only based on TM4. The proportion of children 6-23 months who received foods from 4 or more food groups (TM2 and TM4 were used to calculate 7 food group score).
5: Minimum accepted diet	Proportion of children 6-23 months of age who had at least the minimum dietary diversity (7 food group score>=4) and the minimum meal frequency(TM5>=2 in age 183-274 days or TM>=3 in age 274-730) during the previous day and the proportion of children 6-23 months of age who had at least 2 milk feeding (TM2>=2) and had at least the minimum dietary diversity not including milk feeds (6 food group score>=4) and the minimum meal frequency ((TM2+TM5)>=4) during the previous day.
6: Consumption of iron-rich or iron fortified foods	Proportion of children 6-23 months of age who received an iron-rich food (TM47=1 or TM48=1 or TM410=1) or a food that was specially designed for infants and young children and was fortified with iron, or food that was fortified in the home with a product that included iron (TM3=1) during the previous day.
